# The effects of COVID-19 on the vestibular system

**DOI:** 10.3389/fneur.2023.1134540

**Published:** 2023-03-10

**Authors:** Lena Zaubitzer, Sonja Ludwig, Michelle Berkemann, Beatrice Walter, Frederic Jungbauer, Valentin Held, Stefan C. A. Hegemann, Nicole Rotter, Angela Schell

**Affiliations:** ^1^Department of Otorhinolaryngology, Head and Neck Surgery, University Hospital Mannheim, Medical Faculty Mannheim, University of Heidelberg, Mannheim, Germany; ^2^Department of Neurology, University Hospital Mannheim, Medical Faculty Mannheim, University of Heidelberg, Mannheim, Germany; ^3^Balance Clinic Zurich, Zurich, Switzerland

**Keywords:** vestibular-evoked myogenic potential (VEMP), vHIT, SVV, COVID-19, vestibular system

## Abstract

**Introduction:**

The symptoms and severity of SARS-CoV-2 infection vary greatly across the spectrum, from asymptomatic infection to severe pneumonia with acute respiratory distress syndrome and even death. Dizziness is a frequently reported symptom of SARS-CoV-2 viral infection. However, the extent to which this symptom results from the effect of SARS-CoV-2 on the vestibular system remains unclear.

**Materials and methods:**

In the present single-center, prospective cohort study, patients with a previous SARS-CoV-2 infection underwent a vestibular assessment consisting of the Dizziness Handicap Inventory to assess dizziness during and after infection, a clinical examination, the video head impulse test, and the subjective visual vertical test. When the subjective visual vertical test result was abnormal, vestibular-evoked myogenic potentials were performed. Vestibular testing results were compared to pre-existing normative data of healthy controls. In addition, we performed a retrospective data analysis of patients admitted to hospital presenting with acute symptoms of dizziness who were also diagnosed with acute SARS-CoV-2 infection.

**Results:**

A total of 50 participants have been enrolled. During and after the SARS-CoV-2 infection, women were significantly more likely than men to suffer from dizziness. A significantly reduced semicircular canal or otolith function was not observed in either women or men. Acute SARS-CoV-2 infection was diagnosed in nine patients who presented to the emergency room with acute vestibular syndrome. Six of the patients exhibited acute unilateral peripheral vestibulopathy upon diagnosis. A different patient was diagnosed with vestibular migraine, and two individuals had a posterior inferior cerebellar artery infarct revealed by magnetic resonance imaging.

**Discussion/conclusion:**

Overall, a persisting structural affection of the vestibular system by SARS-CoV-2 seems to be unlikely and could not be confirmed by vHIT, SVV, and VEMPS in our study. It seems possible but unlikely that SARS-CoV-2 induces acute vestibulopathy. Nevertheless, dizziness is a common symptom in patients with COVID-19, which should be taken and worked through seriously.

## Introduction

Dizziness and vertigo are common symptoms in Corona Virus Disease-19 (COVID-19) infections and have been widely described, especially in infections with wildtype Severe Acute Respiratory Syndrome Corona Virus-2 (SARS-CoV-2) virus (1, 2). Various pathomechanisms have been proposed as potential underlying causes for this symptom, ranging from direct neuroinvasion or neuropathy to hypoxia or hypercoagulopathy (3, 4). Among these reasons, autonomic dysfunction has been frequently observed in patients during and after COVID-19 infection (5–7). Additionally, otologic symptoms have been reported to be associated with COVID-19 (8). For example, a recent study by Tan et al. showed worse high-frequency audiometry and reduced otolith and semicircular function as measured by video head impulse test (vHIT) and vestibular-evoked myogenic potential (VEMP) testing (9). However, Tan's study included only relatively young individuals with COVID-19 (mean age, 28.98 years) and a rather small cohort of 26 individuals; the results, therefore, might not be universally transferable. In addition to this study, cases of vestibular neuritis and sudden-onset sensorineural hearing loss after COVID-19 have been described (10–12).

Therefore, it still seems to remain unclear which effect the SARS-CoV-2 virus might have on the vestibular system, both in the context of acute infection as well as in the context of long-term effects. Furthermore, the location of any possible damage that SARS-CoV-2 may cause to the vestibular system needs to be further examined, as the peripheral vestibular end organs or central vestibular structures might both be considered as sites of predilection. The aim of the present study, therefore, was to objectively measure vestibular function in patients with persistent complaints of dizziness after COVID-19 infection. Additionally, we carried out a retrospective review of patients who presented with acute vestibular syndrome (AVS) to our emergency room between September 2020 and March 2022 and who proved to have a positive polymerase chain reaction COVID-19 test with no further symptoms of disease other than vertigo.

## Materials and methods

In our study, which was conducted at a tertiary referral center, we included 50 patients aged 18 years or older with COVID-19 wild-type infection from May to November 2020. COVID-19 diagnosis was confirmed by polymerase chain reaction testing from swab samples. Patients with a previous history of vestibular disorders were excluded from study participation. The study design was an observational, cohort study design based on a convenience sampling method.

For evaluation of vestibular symptoms, subjects filled out the Dizziness Handicap Inventory (DHI) (13) during COVID-19 infection and then at two points of follow-up: at 6 months and again at 9–12 months after COVID-19 infection. Additionally, horizontal vHIT and subjective visual vertical (SVV) testing were performed in all participants 6 months after infection. If SVV testing showed abnormal results, VEMP testing was performed. Moreover, pure-tone audiometry was performed in all patients to rule out a significant hearing loss. If any of the included subjects presented with abnormal laboratory vestibular testing results at the 6-month post-infection visit, vHIT, SVV, and VEMP testing were repeated at the 9- to 12-month follow-up. In addition, vestibular testing results of COVID patients were compared with pre-existing normative data of healthy controls.

Moreover, a retrospective data analysis was performed in patients who presented with AVS at the emergency room between September 2020 and March 2022. Further descriptive patient characteristics were collected on all patients who could be identified who presented with AVS, had no COVID-specific symptoms, but simultaneously had acute COVID-19 infection diagnosed by routinely performed swab samples. The study was approved by the local ethics committee (#2020-549N) and conducted according to the Declaration of Helsinki; all patients gave written informed consent to participate.

### Dizziness handicap inventory

All subjects filled out the DHI questionnaire for evaluation of vestibular symptoms during their COVID-19 infection and again at 6 months and 9–12 months after infection. The DHI is a standardized 25-item self-assessment inventory designed and validated to evaluate the self-perceived detrimental effects imposed by dizziness on private, social, and work life (13). Nine items each evaluate the functional and emotional aspects of dizziness, and the remaining seven items evaluate the physical aspects. Possible answers are “yes,” “no,” and “sometimes”. The calculated point score indicates the severity of the handicap (16–34 points, mild; 36–52 points, moderate; and >54 points, severe). For the purpose of this study, we used the German version of the DHI (14).

### Video head impulse test

Horizontal semicircular canal function was assessed performing vHIT using a video-oculography system (Autronic; Hamburg, Germany) with a high-speed infrared camera and an accelerometer to record head and ocular movement at a sampling rate of 250 Hz (15). The vestibular ocular reflex was calculated during short horizontal head rotations/impulses with aimed head rotations at 100–200/s and 5° and 15° (center to lateral position). Mean gains at 40, 60, and 80 ms as well as correctional saccades were acquired, and a minimum of 15 impulses per side were performed. Results were interpreted as normal if median gain was >0.8 and no reproducible catch-up saccades could be identified.

### SVV and VEMP testing

Laser-induced static SVV (Vertitest, INSTRUMENTATION S.A. DIFRA, Eupen, Belgium) without rotation was performed to screen for otolith dysfunction. The SVV was determined by presenting a laser-induced luminous line in otherwise total darkness and requesting that the patient rotate the line to be in a perceived vertical alignment. SVV was performed 6 times for each side. The examination results were automatically documented and analyzed by the corresponding software. For both sides, median value and the standard deviation were determined. The SVV was considered pathological for each side, if the subjects did not align the SVV within 2° of true vertical (16). If SVV tests showed abnormal results, we performed ocular (oVEMP) and cervical (cVEMP) VEMP testing to further identify and objectify otolith dysfunction. For cVEMP, tone burst stimuli (500 Hz; 125 dB SPL; rise/fall time each 1 ms, plateau 2 ms) with a repetition rate of 5/s were used. Electromyographic changes in the sternocleidomastoid muscle were derived. Responses were averaged over 100 stimuli. The first positive and negative peaks were recorded between 13 and 23 ms and were referred to as p13 and n23 responses. cVEMPs were considered absent if no reproducible p13 and n23 responses could be determined. oVEMPs were recorded with a maximal gaze upward. Tone bursts (4/s, rise/fall time each 1 ms, plateau 2 ms) at 500 Hz were applied with 100 dB nHL. All electromyographic signals were amplified and filtered. Recordings were made with a reproducibility of >90% after 100–200 stimuli. The responses were averaged over 50 stimuli. First negative (n1) and positive (p1) peaks were determined. oVEMPs were considered absent if no defined n1 response could be identified.

### Retrospective data analysis

A retrospective data chart review was performed to additionally identify patients who presented with AVS at the emergency room between September 2020 and December 2021 and were simultaneously tested positive for COVID-19 in the routinely performed swab samples. Patients' demographic and clinical findings were retrospectively analyzed.

### Statistical data analysis

To demonstrate a moderate effect (Cohen's = 0.4) for 2 paired samples, a sample size of 50 has sufficient power (assuming alpha = 0.05 and power = 0.8). This has been verified using the SAS procedure PROC POWER. Data analysis was performed using SPSS version 20.0 (IBM Corp., Armonk, NY, USA) to analyze all data. Unpaired *t*-tests were used for analysis of parametric data; for non-parametric data, the Mann-Whitney *U*-test or Wilcoxon test was performed. DHI and clinical tests were examined for correlation using the Pearson correlation coefficient. If data were missing, the subject in question was excluded. A *p*-value < 0.05 was considered statistically significant for statistical analysis. Numeric variables were reported as median and interquartile range (IQR).

## Results

Fifty patients [23 men (46%) and 27 women (54%)] were enrolled in the study, with an average age of 45 years (±15.44 years). In the course of COVID-19, most patients were treated as outpatients [44 patients (88%)], whereas only 5 patients (10%) were admitted as inpatients, including 2 patients (4%) in the intensive care unit (1 patient did not provide any information about his disease course). All 44 patients who received outpatient treatment considered their infection as “mild”, and 36 patients (72%) reported olfactory and gustatory symptoms. The other patient who received inpatient or ICU treatment described their infection as “severe”. All of them were admitted to hospital due to pneumonia.

As for medication, 11 patients (22%) reported to take hypertension medications, 4 patients (8%) antilipemic medication, 5 patients (10%) antidepressants, and 3 patients (6%) received asthma medications and analgesics.

Vestibular symptoms were mentioned by 23 (46%) patients and started at a mean of 2.96 days after COVID-19 diagnosis. Dizziness was categorized as non-specific by all affected, and 14 participants (28%) described worsening of dizziness when standing. Rotational vertigo was not reported by any of the participants. Three patients had to be excluded, as they had been suffering recurrent spells of dizziness before their COVID-19 diagnosis, bringing the total number of patients to 47 (Table 1).

**Table 1:** Demographic data of 50 COVID-19 patients.

**Demographic data:**	***n* (%)**
**Gender:**	
Male	23 (46)
Female	27 (54)
**Age:**	
20–40ys	23 (46)
41–76	28 (56)
Mean (SD)	45 (15.44)
**Treatment:**	
Outpatient	44 (88)
Inpatient	3 (6)
ICU	2 (4)
No data	1 (2)
**Severity of COVID-19**	
“Mild”	44 (88)
“Severe”	5 (10)
No data	1 (2)
**Symptoms COVID-19:**	
Olfactory dysfunction	15 (30)
Gustatory dysfunction	21 (42)
Pneumonia	5 (10)
Tinnitus	2 (4)
Dizziness	23 (46)
**Quality of dizziness** ^*^ **:**	
Non-specific	23 (100)
Worse when standing	14 (60.9)
Rotational	0 (0)

^*^In patients affected by dizziness (n = 23).

Subjective dizziness was assessed in all 47 patients. The DHI score at diagnosis was 16.04, and 6 months after infection it significantly declined to 6.16 (paired one-side *t*-test, *p* < 0.000). The DHI score still significantly declined after 9–12 months post-COVID-19 infection (paired one-side *t*-test *p* = 0.013) compared with DHI scores during COVID-19. Nevertheless, after 9–12 months, DHI scores slightly (but not significantly; *t*-test *p* = 0.053) increased to 7.87 compared with the 6-month DHI score. Overall, women were more likely to experience dizziness than men (*t*-test, *p* < 0.05) and their symptoms tended to be more severe (DHI score 19.9, compared with 11.1 in men) and were more likely to persist 9–12 months after infection (Table 2).

**Table 2:** DHI results during and after COVID-19: median DHI scores and IQR are shown.

**Median DHI scores (IQR)**	**Overall (*n* = 47)**	**Female (*n* = 24)**	**Male (*n* = 23)**
During COVID	16.04 (10.5–21.5)	19.9 (12.4–23.5)	11.1 (10.5–13.1)^*^
6-month follow-up	6.16 (2.5–7.6)^*^	7.7 (3.6–11.8)	2.3 (0.3–4.4)^*^
9–12-month follow-up	7.78 (3.0–8.1)^*^	9.1 (3.7–12.5)	2.7 (0.2–5.1)^*^

^*^Indicates statistically significant results. Overall DHI scores declined 6 months after infection (paired one-side t-test, p < 0.000) and still were reduced in the 9–12 month follow up (paired one-side t-test, p < 0.013): There was no difference between 6 and 9–12 month follow up (paired one-side t-test, p = 0.053). Women were significantly more affected during and after 6 respectively 9–12 months (paired one-side t-test; p < 0.05).

**Figure 1:**
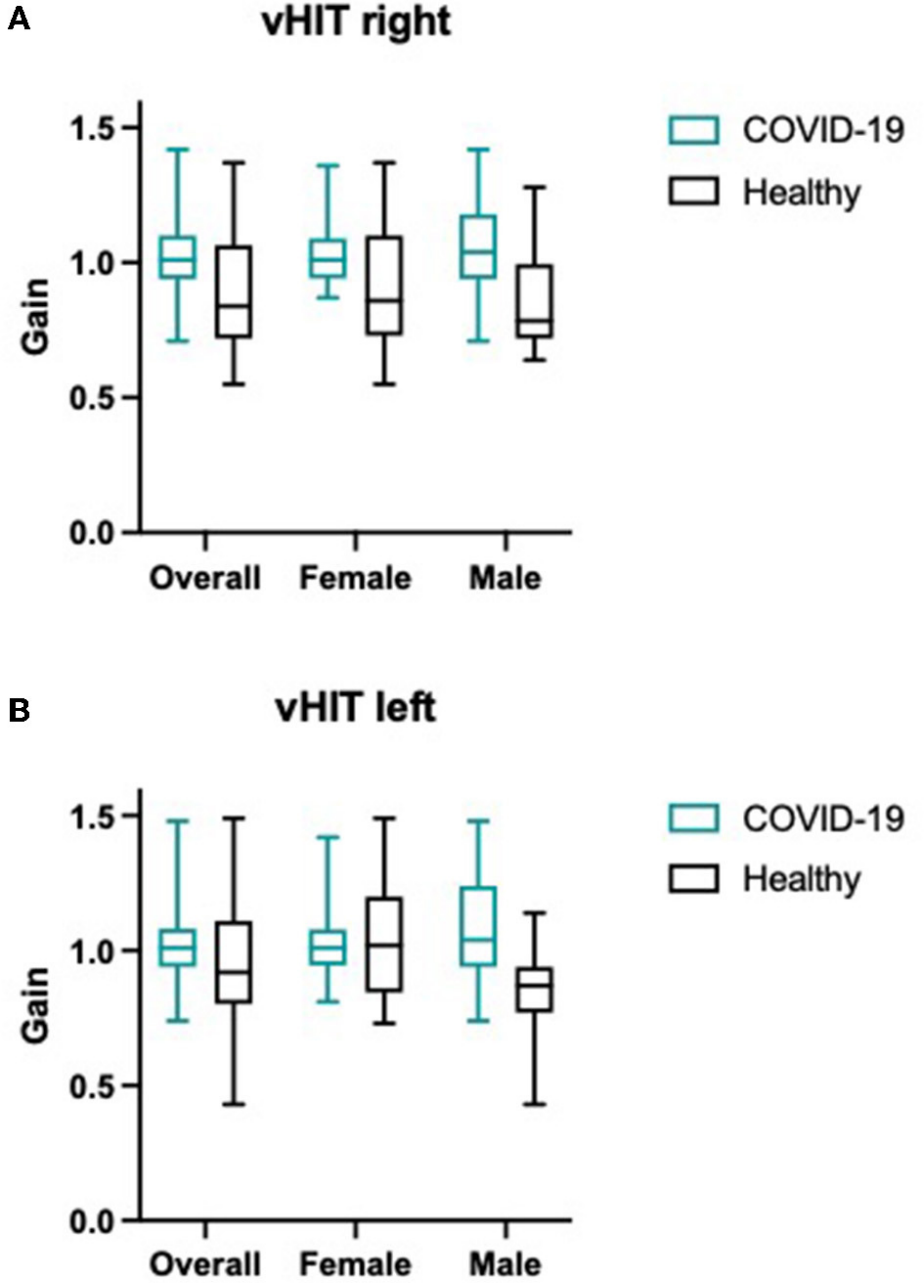
**(A)** vHIT results (median and range) for right and left horizontal canal and **(B)** SVV results (median deviation of true vertical) for overall patients, female and male participants are shown for COVID-19 cohort as well as for control group.

Overall median horizontal gain was 1.01 for the right semicircular canal and 1.01 for the left (*n* = 47, female = 24, male = 23). The SVV testing showed a median deviation from true vertical of 0.9° to the right and 0.3° to the left. All vestibular testing results of COVID patients and pre-existing normative data of healthy controls are shown in Table 3, Figure 1. Only one female patient showed pathological vHIT (bilateral vestibulopathy; horizontal gain reduced to 0.45 right and 0.43 left with clear catch-up overt saccades in both directions) and SVV (4.5° and 5.1° deviation) results. In this patient, cVEMP and oVEMP were performed, but in both tests, no reproducible responses could be measured and were therefore interpreted as “absent”. Interestingly, this patient had a protracted course of infection, requiring intensive care, and also showed bilateral recurrent nerve palsy since the COVID-19 infection. In this patient, 6-month follow-up showed no significant improvement either in vHIT (horizontal gain 0.49 to the right side and 0.43 to the left side, persistent overt saccades) or in VEMP testing. Overall, there was no statistical difference in SVV, VEMP, or vHIT results between genders. COVID patients did not show declined semicircular or otolith function compared to pre-existing data of healthy controls (Table 3, Figure 1). Interestingly COVID-19 patients even showed better vestibular testing results (vHIT right and left: *p* < 0.0001; SVV right: *p*-value 0.0014 and left *p*-value < 0.0001).

**Table 3:** Vestibular testing results.

	**Overall**		**Female**		**Male**	
**vHIT results**	**COVID-19 (*n* = 47)**	**Healthy (*n* = 40)**	**COVID-19 (*n* = 24)**	**Healthy (*n* = 24)**	**COVID-19 (*n* = 23)**	**Healthy (*n* = 16)**
Median gain ± SD (right horizontal canal)	1.01 ± 0.16 (0.71 to 1.42)	0.84 ± 0.21 (0.55 to 1.37)	1.01 ± 0.12 (0.87 to 1.36)	0.86 ± 0.23 (0.55 to 1.37)	1.04 ± 0.19 (0.71 to 1.42)	0.79 ± 0.18 (0.64 to 0.95)
Median gain ± SD (left horizontal canal)	1.01 ± 0.15 (0.74 to 1.48)	0.92 ± 0.21 (0.43 to 1.49)	1.01 ± 0.12 (0.81 to 1.42)	0.97 ± 0.20 (0.73 to 1.49)	1.04 ± 0.18 (0.74 to 1.48)	0.87 ± 0.19 (0.43 to 1.14)
**SVV results**	**(*****n*** = **45)**	**(*****n*** = **20)**	**(*****n*** = **24)**	**(*****n*** = **10)**	**(*****n*** = **21)**	**(*****n*** = **10)**
Median deviation of true vertical (degrees) right	0.9 (−0.2 to 3.9)	0.5 (−0.1 to 1.6)	1.0 (−0.04 to 3.9)	0.6 (−0.1 to 1.5)	0.8 (−0.2 to 2.6)	0.5 (−0.1 to 1.6)
Median deviation of true vertical (degrees) left	0.3 (−1.1 to 2.9)	−0.3 (−1.2 to 0.7)	0.3 (−1.1 to 2.9)	−0.4 (−1.1 to 0.5)	0.2 (−0.5 to 1.8)	0.3 (−1.2 to 0.7)

Above: vHIT results: median gain and standard deviation (SD) as well as range are shown.

Below: SVV results: median deviation of true vertical (in degrees) as well as range are shown.

There were no statistically significant differences between sides or gender (p > 0.05).

Correlation analysis of DHI and median vHIT results did not reveal a significant correlation (Spearman correlation −0.03, p-value 0.8 for right vHIT and 0.1 and p-value 0.4 for left side).

### Retrospective data analysis

In total, 576 patients were identified who presented with AVS at the emergency room between September 2020 and December 2021. *Nine* of those 576 patients presenting with AVS [0.09%; three men, six women; median age, 54 years (range, 29–83 years)] tested positive for COVID-19 by routinely performed swab samples. None of those nine patients mentioned had typical COVID symptoms upon admission to the hospital or had been vaccinated against COVID beforehand. The AVS-patients' clinical findings, diagnoses and subsequent courses of treatment are summarized in Table 4, whereas Table 1 comprises descriptive data of the 50 COVID patients.

**Table 4:** Clinical findings, diagnoses, and subsequent courses of treatment in AVS-case series.

**No**.	**Gender**	**Age**	**Diagnosis**	**Clinical examination and vestibular testing results**	**Subsequent course**
1	w	61	AUV right	HIT right: saccades; left beating horizontal-torsional SN; vHIT with gain reduction 0.65 right; c and oVEMPs absent right side; SVV not performed	Inpatient treatment; iv. corticosteroids; mild COVID symptoms (fever, cough) developed in course, no COVID specific treatment necessary
2	w	45	Vestibular migraine	No SPN documented; normal cerebral imaging; HIT with no saccades; vHIT gain 1.01 (right) and 0.98 (left); SVV < 0 ± 2°	First time episode; outpatient treatment, no COVID symptoms
3	m	29	PICA infarction: Lateral medullary syndrome right	Ipsilateral Horner's syndrome; torsional SP with vertical component and contralesionally beating fast phase; HIT with no saccades; vHIT gain 0.79 (right) and 0.82 (left), no saccades; VEMPs and SVV not performed in acute situation; MRI revealed right dorsolateral medullary infarction	Neurological ICU for monitoring; 2 days after admission patient suffered a pulmonary lung embolism due to deep vein thrombosis; once medically stabilized patient was discharged to subacute rehabilitation; in 3-month follow-up, patient presented with persistent gait instability
4	m	54	AUV left	HIT left: discrete saccades; right beating horizontal-torsional SN; vHIT with gain reduction 0.76 left; VEMPs not performed; SVV with 3.9° deviation toward the lesion side	Inpatient treatment, iv. corticosteroids; no COVID symptoms
5	w	83	PICA infarction right	Gaze-changing SN, HIT with no saccades; vHIT and VEMPs not performed due to lethargic general condition	Neurological ICU for monitoring; 3 days later CT revealed posterior fossa edema and obstructive hydrocephalus, requiring placement of an external ventricular drain (EVD); pulmonary deterioration another 2 days later and COVID ARDS
6	w	51	AUV right	HIT right: distinct overt saccades; left beating horizontal-torsional SN; vHIT right: with gain reduction to 0.55, covert and overt saccades; cVEMP normal, oVEMP absent right side; SVV not performed	Outpatient treatment, no COVID symptoms
7	w	38	AUV right	HIT right: saccades; left beating SN; vHIT right confirmed gain reduction to 0.62; reduced bithermal caloric response on the right side; normal cerebral imaging	Outpatient treatment, no COVID symptoms
8	m	63	AUV left	HIT left: questionable saccades; right beating horizontal-torsional SN; vHIT with gain reduction 0.71 left; cVEMP left normal; reduced bithermal caloric response	Inpatient treatment, iv. corticosteroids: mild COVID symptoms (fever, headache, sore throat) developed in course; no COVID specific treatment necessary
9	w	45	AUV right	Left beating SN; vHIT with gain reduction (0.58) and covert and overt saccades; normal cerebral imaging; SVV with 5.7° deviation toward the lesion side	Inpatient treatment, iv. corticosteroids: mild COVID symptoms (muscle pain, runny nose) developed in course; no COVID specific treatment necessary

## Discussion

Dizziness is a common symptom in patients with COVID-19 infection (2). Numerous reports describe audio-vestibular symptoms associated with COVID-19 (17–19). Nevertheless, even after more than 2 years of the pandemic, the etiopathogenesis of the underlying cause of dizziness remains unclear. It is conceivable and hypothesized that there is a direct effect on the cochlear and vestibular peripheral end organs and/or a neural irritation in the sense of a neuropathy or a virus-mediated immune response. The former is favored by the fact that SARS-CoV-2 viral proteins were detected in cranial nerves originating from the lower brainstem and in brainstem cells (20). Blood vessels, nerves, and the meninges also have been proposed as possible entry routes for the virus (21, 22). To better understand the manifestations of dizziness associated with COVID-19 infection, we combined a retrospective data analysis with a prospective study design.

In our prospective study, we could confirm that patients suffer from dizziness during infection, as DHI score was significantly elevated compared with our two follow-ups, 6 months later and then 9 to 12 months later. Moreover, almost 50% of the participants complained about dizziness, which occurred in a median of 2.96 days after infection. This result is comparable with other studies published recently (23). Women were significantly more often affected than men with dizziness during and after infection, and their DHI scores were significantly higher. This finding does not seem surprising, as women in general are affected more often by vestibular disorders and dizziness-related reduced of quality of life (24, 25).

In contrast to the subjective DHI and self-assessment, vestibular testing did not reveal any significant hypofunction in our study population. Hence, SARS-CoV-2 does not appear to cause long-term hypofunction or dysfunction of the vestibular system, as normal vestibular function was measured by vHIT and SVV testing. COVID patients did not show reduced vestibular function compared to healthy controls. Normal gain values were assessed, and no pathological saccades were seen in all but one patient. Interestingly, the existing literature is inconsistent, which is confirmed especially in two recent meta-analyses (22, 26). The study situation is difficult to compare, as most studies only collect subjective or objective parameters and differ greatly in their methodology. A recent study by Tan et al. describes significantly lower gains compared with healthy controls. However, it must be emphasized that the gains of the COVID group are still in the normal range, and it is questionable whether a small cohort of 26 individuals has sufficient sample size to generalize the findings (9). The same criticism applies to the study by Demir et al. that examines audio-vestibular function in a pediatric COVID collective comprising 35 children age 9–15 years (27). In our cohort, we did not detect overall reduced semicircular canal function or otolith dysfunction. Only one patient presented with bilateral vestibulopathy. Interestingly, this patient had a protracted course of infection, requiring intensive care, and showed bilateral recurrent nerve palsy since the COVID-19 infection. The patient's follow-ups did not show any significant improvement neither in terms of her vestibular system, nor the vocal cords. Usually, in patients with cranial nerve involvement, COVID-19 infections seem to be mild (28), which was not the case for the patient included in this study. However, it remains unclear, whether this is a viral- or drug-induced cranial nerve neuropathy, as the patient underwent intensive care and antiviral therapy.

In the retrospective part of our study, we identified 6 patients with acute peripheral vestibulopathy and PCR testing. Three of them developed COVID-typical symptoms, whereas three of them remained asymptomatic. Uni- or bilateral peripheral vestibulopathy have been described after COVID-19 infection as well as after vaccination (12, 29, 30). Nevertheless, in a recent prospective study by Gerb et al. an increase of central or peripheral vestibular syndromes after COVID-19 vaccination was not observed (31) and Charpiot et al. also did not find a correlation between AVS and COVID-19 (32). In conclusion, it can only be assumed but not proven that SARS-CoV-2 might cause acute vestibulopathy. In our case series, we also identified two patients with posterior circulation stroke a number which seems to be rather large in this small cohort consisting of eight patients. The predominant cause of PICA infarcts generally is an arterial-arterial thrombo-embolism from extracranial large arteries followed by cardio-embolism or in-situ disease (33). The relatively large number of PICA infarctions might be attributable to the systemic effects of COVID-19, particularly possible COVID-related hypercoagulopathy (34, 35). The triggering of coagulopathy in COVID-19 seems to be linked to alveolar damage resulting in an inflammatory storm with activated production of inflammatory cytokines including IL-6, resulting in generation of pro-coagulative factors and damage to endothelium as a prerequisite for thrombosis or arterial to arterial thrombo-embolism (36, 37).

A predilection for posterior infarcts in COVID-19 has also been described by Ahmed et al. (38). The authors hypothesize, that also the anatomical vascular peculiarity and variations can pose a more viable risk to isolated PICA lesions (38).

Nevertheless, due to the pandemic status of COVID-19, AVS and COVID-19 can also be merely a coincidence.

Overall, a structural affection of the vestibular system by SARS-CoV-2 seems to be unlikely and could not be confirmed in our study. Hence, there must be other possible explanations why individuals with COVID-19 experience dizziness: hyperventilation due to (subjective) dyspnea for example, can lead to dizziness and light-headedness which is often seen in patients with anxiety disorders (39). Cardiovascular or orthostatic factors, especially in combination with fever and a potential hypovolemia might also lead to dizziness. At the same time, autonomic dysfunction (i.e., orthostatic hypotension) is also a frequently observed symptom in COVID-19 patients (5–7) and has to be taken into consideration, especially as the dizziness was categorized as non-specific and worse in standing position. Moreover, a somatoform, stress-related component needs to be considered. Also, or precisely because the origin of dizziness in patients with COVID-19 remains unclear, a structured otological and neurological assessment should take place.

Our study has limitations that need to be mentioned. With only 50 patients, our sample size is rather small, although statistically sufficient. Additionally, due to restrictions imposed by hospital hygiene, vestibular laboratory testing was not allowed during the actual COVID-19 infection, but only after the patients recovered; this is a common limitation that has also been described by Fancello et al. (22). However, there is a controversial discussion on how the DHI correlates with objective tests: While Yip et al. found no correlation between balance testing and DHI (40), there are several studies describing a moderate correlation (41, 42).

An even more differentiated objective vestibular assessment would have been desirable, including head tilt or off-axis SVV *and* o- and cVEMP testing in all patients and ideally baseline testing even prior to the infection. A longer follow-up period and routinely performed vestibular tests especially during infection and 6, 9-, and 12-months, respectively, would have also been desirable not only to detect even the subtlest vestibular hypofunctions, but also to identify patients with vestibular affection which may have already been recovered after 6 months.

Moreover, only patients with wildtype infection were included; therefore, our results only reflect the effect of wildtype COVID-19 infection and potentially might not be generalizable for all variants. To date, no study has investigated the impact of different SARS-CoV-2 variants on the sensory system. It would be interesting to investigate whether the different variants have different influences on the sensory system overall and on the vestibular system in particular.

## Conclusion

Overall, a persisting structural affection of the vestibular system by SARS-CoV-2 seems to be unlikely and could not be confirmed by vHIT, SVV and VEMPS in our study. It seems possible but unlikely that SARS-CoV-2 induces acute vestibulopathy. Nevertheless, dizziness is a common symptom in patients with COVID-19, which should be taken and worked through seriously.

## Data availability statement

The raw data supporting the conclusions of this article will be made available by the authors, without undue reservation.

## Ethics statement

The studies involving human participants were reviewed and approved by Ethik-Kommission II der Universität Heidelberg Medizinische Fakultät Mannheim Theodor-Kutzer-Ufer 1-3 68167 Mannheim. The patients/participants provided their written informed consent to participate in this study.

## Author contributions

LZ and SL recruited patients, performed statistical analysis, provided conceptual support, and wrote the manuscript. MB recruited patients and performed statistical analyses. BW and FJ recruited patients and helped interpreting the results. VH and SH supported writing and finalizing the manuscript from a neurological perspective. NR recruited patients and advised in writing the manuscript. AS provided conceptual support, supervised the study, and helped writing and finalizing the manuscript. All authors contributed to the article and approved the submitted version.
